# Microbiological Profiles, Antibiotic Susceptibility Patterns and the Role of Multidrug-Resistant Organisms in Patients Diagnosed with Periprosthetic Joint Infection over 8 Years: Results from a Single-Center Observational Cohort Study from Romania

**DOI:** 10.3390/microorganisms13051168

**Published:** 2025-05-21

**Authors:** Serban Dragosloveanu, Rares-Mircea Birlutiu, Bogdan Neamtu, Victoria Birlutiu

**Affiliations:** 1 Department 14-Orthopedics, Anaesthesia Intensive Care Unit, Faculty of Medicine, “Carol Davila” University of Medicine and Pharmacy, 050474 Bucharest, Romania; serban.dragosloveanu@umfcd.ro; 2 Foisor Clinical Hospital of Orthopedics, Traumatology, and Osteoarticular TB, 021382 Bucharest, Romania; 3 Faculty of Medicine, Lucian Blaga University of Sibiu, 550169 Sibiu, Romania; 4 Pediatric Research Department, Pediatric Clinical Hospital Sibiu, 550166 Sibiu, Romania; 5 Bioinformatics and Biostatistics Department, University of Louisville, Louisville, KY 40202, USA; 6 County Clinical Emergency Hospital, 550245 Sibiu, Romania

**Keywords:** prosthetic joint infections, microbial etiology, antibiotic susceptibility tests, antimicrobial empirical treatment, multidrug-resistant organism, patterns, hip, knee

## Abstract

This study examines temporal patterns in pathogens isolated from prosthetic joint infection (PJI) cases and antimicrobial resistance patterns at a Romanian orthopedic center. We have conducted a retrospective cohort study that included 674 patients undergoing hip or knee replacement revision surgery between January 2016 and December 2023. From these, 102 confirmed PJI cases requiring surgical intervention were selected for analysis. We isolated 27 microorganisms from acute PJI cultures and 82 from chronic PJIs. *Staphylococcus epidermidis* (33 cases, 30.3%; 95% CI 22.0–40.3) was the predominant pathogen, with coagulase-negative *Staphylococci* (22 cases, 20.18%; 95% CI 0.9–41.3) and Enterobacteriaceae (13 cases, 11.9%; 95% CI 6.4–18.3) also prevalent. Methicillin resistance was identified in 43.6% of coagulase-negative staphylococci and 45.5% of *Staphylococcus aureus* isolates. All Gram-positive isolates remained susceptible to vancomycin, linezolid, and tigecycline. Among Gram-negative bacilli, *Klebsiella oxytoca* and *Proteus mirabilis* showed resistance to third-generation cephalosporins, with phenotypic profiles suggestive of extended-spectrum β-lactamase (ESBL) production. All *Escherichia coli*, *Enterobacter* spp., and *Citrobacter freundii* strains were fully susceptible to tested agents, while *Pseudomonas aeruginosa* exhibited reduced susceptibility to ciprofloxacin, aztreonam, and imipenem. Among the isolated strains, 47 were multidrug-resistant (MDR), with *Staphylococcus aureus* accounting for the highest MDR count, including methicillin resistance. The distribution of microorganism types and MDR strains remained consistent throughout the study period, with no significant association between infection type and MDR strain presence or between infection site and microorganism presence except for a strong association between MDR strains and the type of microorganism (*p* < 0.05). The microbial profile and resistance patterns in PJIs have remained stable over eight years. Our observations do not suggest that MDR PJIs are more commonly acute cases, contrary to what has been highlighted in previous reports. The ongoing prevalence of MDR strains underscores the importance of targeted antimicrobial treatments based on local susceptibility profiles.

## 1. Introduction

Bacterial biofilms have emerged as significant contributors to global health issues, exhibiting high antibiotic resistance, interactions with the host’s immune defense systems, and responses to numerous external factors [1]. These biofilms are commonly found on hospital instruments, body tissues, industrial and food processing units, and within natural environments. Nearly all bacteria have the capability to form them [1]. Biofilm-related infections (BRIs) cover a wide range of infections caused by biofilm formation. The most prevalent BRIs include catheter-associated urinary tract infections, with other types being central line-associated bloodstream infections, BRIs related to fixed braces, fracture-related infections, and periprosthetic joint infections [2,3,4]. Bacteria are classified into two categories: planktonic, existing as single-celled structures, and biofilms, organized in multicellular aggregates as sessile structures. Despite advances in research, a consensus on the biofilm definition remains elusive. However, biofilms are commonly described as coherent clusters of bacterial cells embedded in a biopolymer matrix. This matrix increases their tolerance to antimicrobials compared to planktonic cells and provides resistance against the host’s antimicrobial defenses [5,6]. Zhao A et al. define a biofilm as fixed microbial communities encased in extracellular polymeric substances [1].

The number of primary total joint arthroplasty procedures is projected to rise significantly in the next decade, which will consequently increase the demand for revision arthroplasty procedures. By 2030, the incidence of revision total hip arthroplasty and total knee arthroplasty is anticipated to grow by 43 to 73% and 78 to 182%, respectively [7]. Despite advancements in primary total knee arthroplasty that have eliminated failures due to polyethylene wear, infections remain a primary cause for revisions [8,9]. Periprosthetic joint infections (PJIs) represent a significant complication in orthopedic surgery, carrying a substantial socioeconomic impact. As the frequency of primary arthroplasty procedures continues to climb, the significance of managing implant-associated infections will similarly escalate [10,11,12]. The results of a recent study conducted in our institution regarding the causes for total knee arthroplasty revision, performed over a period spanning from 2003 to 2023, highlight the rates of early and chronic periprosthetic joint infection to be 12.55% and 34.01%, respectively [13].

To potentially cure PJIs, a combination of antimicrobial therapy and surgery is essential [10,14,15,16,17,18,19,20]. The standard practice involves initiating broad-spectrum antimicrobial agents following the collection of intraoperative cultures. The critical role of appropriate initial empirical antimicrobial therapy in the success of treating infections is well-documented [21,22]. Upon identifying the pathogen and obtaining antimicrobial susceptibility results, a more targeted narrow-spectrum antibiotic regimen is selected to continue treatment [10,14,15,16,21,22]. Nevertheless, a considerable proportion of patients (5–35%) yield negative culture results [10,14]. In these cases, selecting an appropriate empirical antimicrobial therapy becomes crucial, albeit challenging, given that treatment may need to be extended for several weeks to months to effectively cure a PJI [10,14].

Most prosthetic joint infections are caused by Gram-positive bacteria, with staphylococci and streptococci representing 65% to 85% of cases. Although Gram-negative organisms are less frequently identified, occurring in only 6% to 23% of cases, their significance cannot be understated. This is due to the requirement for broader antibiotic coverage and their association with treatment failures [23]. In their 2016 study, Benitio N. et al. reported an increase in the proportion of PJIs caused by aerobic Gram-negative bacilli. Additionally, this group of authors noted a rise in the incidence of PJIs attributable to fungi [24].

The only published data on local epidemiology trends of prosthetic joint infections (PJIs) in Romania comes from Roman M. et al. [4]. Their work significantly enhances our understanding of the microorganisms causing PJIs in this region. They analyzed numerous cases, finding that most infections were attributed to a single pathogen, with 55 cases identified as being primarily caused by *Staphylococcus aureus*. However, their statistical analysis did not reveal any significant linear trends in PJIs caused by Gram-positive aerobic or microaerophilic cocci, Gram-negative aerobic bacilli, or multidrug-resistant bacteria. Understanding biofilm formation on non-living surfaces is crucial for treating implant-associated bone and joint infections, where successful treatment involves both surgical management and antibiotic therapy. Key to this process is identifying the pathogen and determining its antimicrobial susceptibility through testing (AST), yet local data on these aspects remains limited. Moreover, there is a need for ongoing research to tailor empirical antibiotic therapy to local microbial profiles and for surveillance to optimize PJI management strategies globally.

This paper investigates recent patterns in the microorganisms isolated from periprosthetic joint infection (PJI) cases treated at a leading orthopedic surgery center in Romania. The analysis encompasses both cases originating within the center and those referred from other institutions, offering a detailed examination of different types of PJIs. Our goal was to enrich the current understanding of PJI at both the European and international levels. Furthermore, we aimed to explore shifts in the prevalence of antimicrobial-resistant organisms within PJIs considering potential differences between acute and chronic PJIs. Recent reports suggested more MDR in the acute PJIs [25].

## 2. Materials and Methods

### 2.1. Study Design

A single-center observational cohort study was conducted at the “Foisor” Clinical Hospital of Orthopedics, Traumatology, and Osteoarticular TB in Bucharest, Romania, recognized as a center of excellence for orthopedic surgeries, a national referral facility with high surgical volume, recognized for its specialization in complex orthopedic procedures, including joint arthroplasty and the management of musculoskeletal infections, complex spine surgeries, but not only this. The study received ethical approval from the hospital’s ethics committee (file number 13449/07-12-2023) and underwent review by the institutional management board, securing its approval before patients’ inclusion. The authors accessed the electronic medical records and data from 8 December 2023 to 31 December 2023. The authors had access to information that could identify individual participants during data collection.

A standardized diagnostic system was implemented and used to assess all patients who underwent surgical intervention for the revision of a joint prosthesis to determine the cause of implant failure.

### 2.2. Study Population

We retrospectively selected all consecutive patients aged over 18 years who were hospitalized between January 2016 and December 2023 and underwent joint arthroplasty revision surgery due to periprosthetic joint infections. We excluded cases with positive bacterial cultures from specimens harvested during the second stage of the two-stage revision surgery. Detailed patient information was extracted using a standardized electronic form directly from the medical records, ensuring all data for the enrolled patients were comprehensive and available. This information was independently reviewed by two authors to verify its accuracy and reliability, ensuring consistency and robustness in the data used for analysis.

### 2.3. Laboratory Studies

A standardized diagnostic approach was applied to ensure consistent and thorough evaluation across cases. It encompassed the standardized collection of at least six intraoperative tissue samples. Specifically, two were designated for histopathological examination of the periprosthetic membrane, while the remainder were sent for bacterial cultures in the microbiology laboratory. For the sonication of retrieved implants, we adhered to strict sterility protocols. This process involved adding saline solution to implants within sterile containers under laminar airflow conditions in the operating room, then processed in a Class II microbiological safety cabinet. Containers were double-packed and sterilized according to manufacturer specifications. Implants were sonicated within 20–30 min using an ultrasound bath at 42 kHz frequency and 0.22 W/cm^2^ power density. The sonication fluid was vortexed, and 50 mL centrifuged at 2500 rpm for 5 min; a precipitate was inoculated and a count exceeding 50 CFU/mL indicated positive cultures. Sonication was introduced as a standard diagnostic method in our institution in late 2015.

Periprosthetic tissue samples were individually homogenized in 1 mL thioglycolate broth before inoculation into culture media. Synovial fluid, aspirated preoperatively or intraoperatively, was also cultured in various media. All biological samples underwent incubation under aerobic, anaerobic, and high-concentration CO_2_ conditions using Schaedler anaerobe broth, a Sabouraud plate, a MacConkey agar plate, glucose broth, lactose broth, and thioglycolate broth at 35–37 °C, with additional media employed as necessary. All specimens were inoculated on aerobic and anaerobic culture media and a 14-day period of incubation was implemented as a standard. Bacterial identification was conducted using a VITEK 2 Compact analyzer (bioMérieux, Marcy-l’Étoile, France).

Since 2020, in all cases where clinical and intraoperative findings suggested infection, but cultures remained negative after 48 h, or where rapid identification of atypical or fastidious organisms was necessary, we implemented a molecular diagnostic protocol based on multiplex PCR amplification using biotinylated primers HybriSpot (vitro master diagnostica, Madrid, Spain). This was followed by reverse hybridization onto membranes containing specific pathogen probes. Notably, in all instances, the molecular assay results were concordant with those obtained from conventional cultures.

Antibiotic susceptibility testing (AST) was performed using fully or semi-automated systems, most frequently the MICRONAUT system (MERLIN Diagnostika GmbH, Bornheim, Germany). AST was conducted on pure bacterial colonies suspended in sterile NaCl to achieve a turbidity of 1.8–2.2 McFarland units. Minimum inhibitory concentrations (MICs) were interpreted according to the breakpoints established by the European Committee on Antimicrobial Susceptibility Testing (EUCAST). We streamlined the results and reported only the AST for the most frequently isolated strains. The AST results were categorized as susceptible (S), intermediate (I), or resistant (R) based on the MIC values. A polymicrobial prosthetic joint infection (PJI) was identified by the presence of multiple microorganisms in tissue biopsies, sonication, or synovial fluid cultures. Our capacity to analyze cultures extended to both weekdays and weekends.

### 2.4. Study Definitions and Classification

A culture has been taken into consideration positively upon the identification of an isolate, marking the first day of growth. The diagnosis of a periprosthetic joint infection followed the 2021 European Bone and Joint Infection Society (EBJIS) criteria if diagnosed after 2022 [26], or the criteria defined by Parvizi et al. [27] for diagnoses prior. The criteria included the following: (i) the presence of a sinus tract communicating with the prosthesis/joint or visible implant, (ii) the isolation of the same microorganism from two or more cultures, or (iii) the isolation of a microorganism in intraoperative cultures with additional infection evidence (such as positive histology, purulence, elevated serum erythrocyte sedimentation rate, C-reactive protein or D-Dimer levels, and elevated synovial white blood cell count or positive leukocyte esterase). This approach was adopted to reflect the evolving standards implemented in our institution during the study period. Each case was diagnosed based on the criteria that were active at the time of diagnosis. No case met one definition but failed another.

Following our previously published studies [4,19], we have taken into consideration two classifications, the one proposed by Zimmerli et al. [28] and the PRO-IMPLANT Foundation’s Pocket Guide to Diagnosis & Treatment of PJI, Berlin, Germany, coordinated by N. Renz and A. Trampuz [29]. Due to the limited number of patients, we adopted a simpler classification from the PRO-IMPLANT Foundation’s Pocket Guide to Diagnosis & Treatment of PJI, Berlin, Germany, coordinated by N. Renz and A. Trampuz. This guide, consistent with national and international recommendations, classifies periprosthetic infections as either acute or chronic (perioperative/hematogenous or per continuitatem). The classification of bacterial isolates as MDR was based on their resistance to at least one agent in three or more antimicrobial categories in accordance with international definitions [29].

### 2.5. Statistical Analyses

We performed the statistical analysis using the IBM SPSS Statistics^®^ version 29 software. Continuous variables were summarized as medians and mean and categorical variables as percentages of the total sample for that variable. Overall percentages of culture-positive PJIs were calculated and estimated with a 95% CI. Pearson’s Chi-Square test was applied for the analysis of associations within categorical data. The Chi-Square test was employed to determine statistically significant differences in the proportions of infection caused by specific microorganisms/groups of organisms or the MDR patterns over time. A significant level of *p* ≤ 0.05 was used for all statistical tests.

## 3. Results

### 3.1. Demographics

Between January 2016 and December 2023, our hospital performed revision surgeries on 674 hip or knee joint replacements. During this period, 556 adult patients were diagnosed with aseptic loosening of an endoprosthesis. Additionally, we identified 89 episodes of periprosthetic joint infections from an equal number of culture-positive cases. For our final analysis, we excluded nine cultures deemed contaminants and seven cases with positive cultures from specimens collected during the second stage of two-stage revision surgeries. Thirteen patients had culture-negative PJIs. Ultimately, 102 confirmed PJI cases, which received treatment through debridement, antibiotics, and implant retention procedures, or underwent one-stage or two-stage revision surgery, were analyzed.

The patient enrollment data reveal a gradual progression over the years, starting with four enrollments in 2016 and increasing to six enrollments in 2017, followed by a decrease to three enrollments in 2018. A notable rise was observed in 2019, with 17 patients enrolled, before stabilizing at 10 enrollments in 2020 and 7 enrollments in 2021. In 2022, the number of enrolled patients increased to 26, culminating in a peak of 33 enrollments in 2023.

Table 1 outlines the characteristics of the study’s participants. Of these, 53 (51.96%) were male. The average body mass index was 30.59 kg/m^2^ (range: 18.20–42.00), with a mean age of 67.08 years (standard deviation ± 8.848). The median American Society of Anesthesiologists (ASA) score was 2 (range 1–4), and the median Charlson Comorbidity Index was 3 (range 0–6). Ninety-four patients had at least one comorbidity. The most common primary indication for prosthesis implantation was osteoarthritis (n = 60, 58.82%), followed by femoral neck fracture (n = 12, 11.76%), avascular necrosis (n = 9, 8.82%), and rheumatoid arthritis (n = 4, 3.92%).

Out of 102 PJIs, we categorized 24 as acute or acute hematogenous and 78 as chronic. Specifically, there were 19 acute and 5 acute hematogenous cases. Due to the limited patient numbers, we combined both acute categories for analysis to enhance the statistical power and accuracy of our findings. Among these PJIs, 55 (53.92%) were associated with hip prostheses and 47 (46.07%) with knee prostheses. Revision implants were involved in 14 cases (13.72%). The average time from prosthesis implantation to revision surgery for PJIs was 39.39 months, ranging from 7 days to 25 years. Additionally, 22 patients were referred to our hospital for their PJI cases.

### 3.2. Microbiological Studies and Patterns

We isolated 27 microorganisms from the 24 acute/acute hematogenous PJI cultures. The 78 chronic PJI cultures yielded 82 isolated microorganisms [1,2].

According to our results, 80 (73.4%; 95% CI 64.1–81.7) episodes of PJIs were caused by Gram-positive aerobic or microaerophilic cocci and 14 (12.8%; 95% CI 7.3–19.3) by Gram-negative aerobic bacilli. Overall, *Staphylococcus epidermidis* was the most common isolated pathogen in our series 33 (30.3%; 95% CI 22.0–40.3), followed by other coagulase-negative staphylococci (CoNS) 22 (20.18%; 95% CI 0.9–41.3), *Enterobacteriaceae* 13 (11.9%; 95% CI 6.4–18.3), and *Staphylococcus aureus* 11 (10.1%; 95% CI 4.6–16.5).

A microbiological diagnosis was obtained in 89 cases: 10 cases in 2016–2017, 20 in 2018–2019, 17 in 2020–2021, and 62 in 2022–2023. A significant variation in the proportion of cases with a microbiological diagnosis using our diagnostic method was not observed during the study period (*p* = 0.119). Seven cases of PJIs were polymicrobial, and four of them were chronic cases of PJIs caused by *Staphylococcus epidermidis* + *Enterobacter cloacae*, *Staphylococcus epidermidis* + *Staphylococcus simulans*, *Enterococcus faecium* + *Proteus mirabilis*, and *Proteus* spp. + *Coagulase-negative Staphylococcus* and three cases of acute PJIs caused by (*Staphylococcus epidermidis* + *Staphylococcus capitis*, *Pseudomonas aeruginosa* + *Candida albicans*, *Proteus mirabilis* + *E. coli*). Also, no significant patterns in the proportion of polymicrobial PJIs over the study period were observed (*p* = 0.682).

Among the analyzed cases, 13 cases (11.9%; 95% CI: 5.5–18.3) were culture-negative.

Table 2 represents the lists of causative microorganisms of PJIs during the study period (2016 through 2023). Gram-positive aerobic or microaerophilic cocci were the most common group of isolated organisms, followed by Gram-negative aerobic bacilli.

We further conducted a biennial analysis of the microorganism types isolated from PJIs, excluding cases caused by *Candida* spp. (due to the fact that one strain of *Candida tropicalis* was isolated in 2019 and one strain of *Candida albicans* was isolated in 2022) and Gram-negative anaerobic bacilli (due to the fact that one strain of Gram-negative anaerobic bacilli was isolated in 2020). This analysis included polymicrobial infections, cases without bacterial growth, Gram-negative aerobic bacilli, and Gram-positive aerobic or microaerophilic cocci. The years of diagnosis were categorized into four subcategories: 2016/2017, 2018/2019, 2020/2021, and 2022/2023.

**Figure 1:**
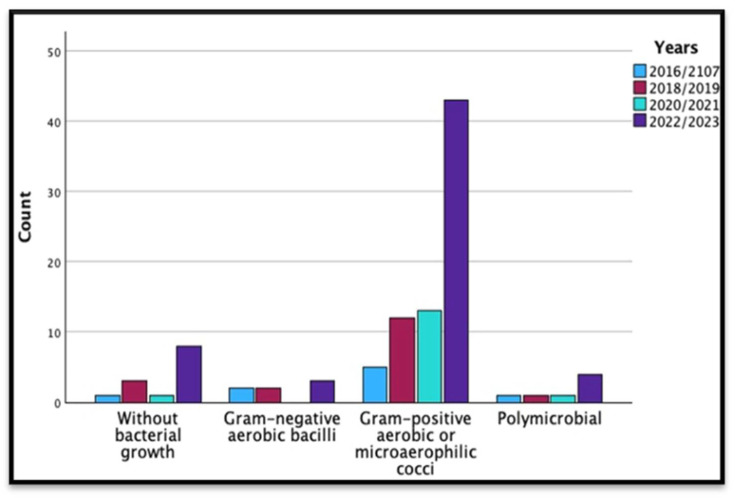
Patters in the microbial etiology of PJIs during the study period by years in subcategories.

The statistical analysis revealed no significant changes in the distribution of microorganism types over the studied periods. High *p*-values (*p* = 0.680) suggested that variations in microorganism counts were likely attributable to chance, indicating no significant trend or pattern. Further details on the analysis are presented in Table 3, and Figure 1.

**Figure 2:**
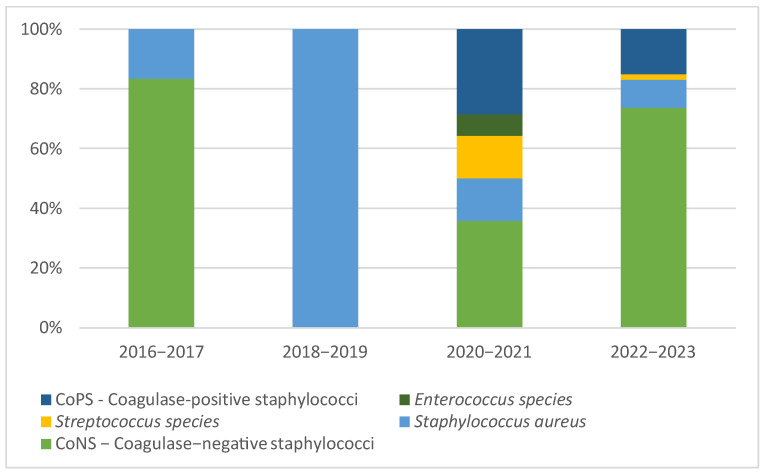
Patterns in the microbial etiology of PJIs: distribution of Gram-positive aerobic or microaerophilic cocci during the study period.

**Figure 3:**
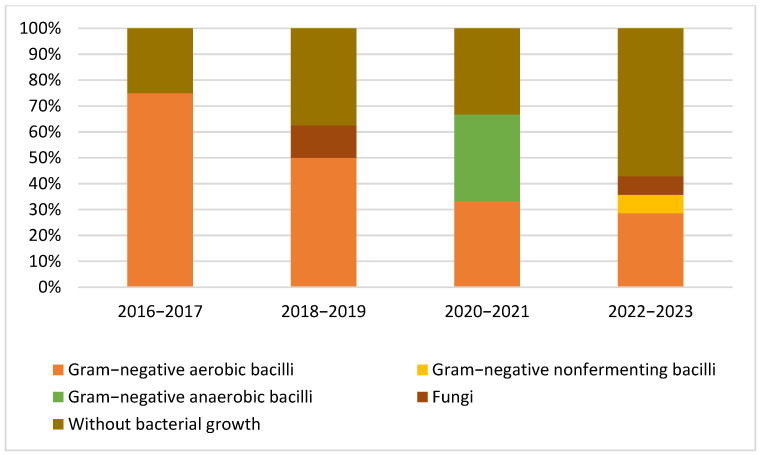
Patterns in the microbial etiology of PJIs: distribution of Gram-negative aerobic bacilli, Gram-negative nonfermenting bacilli, Gram-negative anaerobic bacilli, cases without bacterial growth, and fungi during the study period.

Figure 2 and Figure 3 illustrate the eight-year patterns in the microbial causes of PJIs, showcasing the distribution of various organisms: Gram-positive aerobic or microaerophilic cocci, Gram-negative aerobic bacilli, Gram-negative nonfermenting bacilli, Gram-negative anaerobic bacilli, cases without bacterial growth, and fungal infections.

Table 4 highlighted the results of a crosstabulation between the identified microorganisms and the type of periprosthetic joint infection. Acute or acute hematogenous infections were associated with 24 cases, while chronic infections accounted for 78 cases. The most common microorganism associated with infection was *Staphylococcus epidermidis*, which appeared 33 times, predominantly in chronic infections (27 cases). Coagulase-positive staphylococci were next, with nine isolated strains, majorly in chronic PJIs (seven cases). Methicillin-resistant *Staphylococcus aureus* and *Staphylococcus hominis* had five isolated strains, mostly in chronic PJIs. Microorganisms like *Candida albicans*, *Citrobacter freundii*, *E. coli*, and several others were solely associated with acute infections in our study.

Conversely, *Staphylococcus saprophyticus* and several *Enterobacter* species were exclusively associated with chronic PJIs. Some microorganisms, such as *Proteus mirabilis*, *Staphylococcus capitis*, and *Staphylococcus lugdunensis*, were isolated in both acute and chronic PJIs, with a slight leaning towards chronic cases of infections. Cases without bacterial growth were predominantly chronic (12 out of 13 instances). Overall, there was no significant relationship between the type of microorganism and the type of infection when considering the data as a whole (as indicated by Pearson’s Chi-Square (value was 33.759 with 28 degrees of freedom, and the asymptotic significance (*p*-value) was 0.209) and Likelihood Ratio tests (value was 35.552, 28 degrees of freedom, and a *p*-value of 0.154)).

The crosstabulation analysis involving microorganisms, types of infection, and year of diagnosis data (Table A1) revealed that Pearson’s Chi-Square test *p*-values from 2016 to 2023 did not indicate significant associations (*p* > 0.05), except for 2022 with the Linear-by-Linear Association showing significance (*p* = 0.013). Similarly, a significant Likelihood Ratio in 2023 (*p* = 0.015) implied a noteworthy association for that year. The year 2022 presented a tendency towards statistical significance *p*-value (0.097).

**Figure A1:**
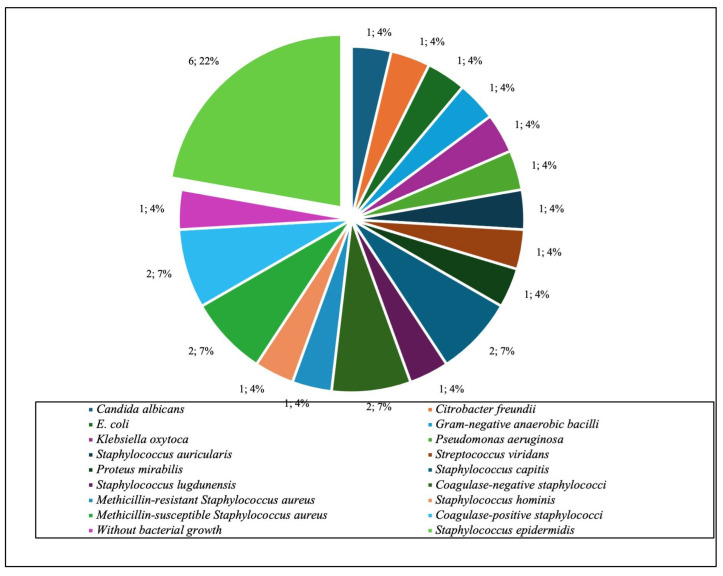
Isolated strains from acute/acute hematogenous PJIs.

Figure A1 illustrates the microorganisms isolated from acute and acute hematogenous PJI cases. *Staphylococcus epidermidis* emerges as the predominant strain, accounting for 22% of these cases, with six strains isolated.

**Figure A2:**
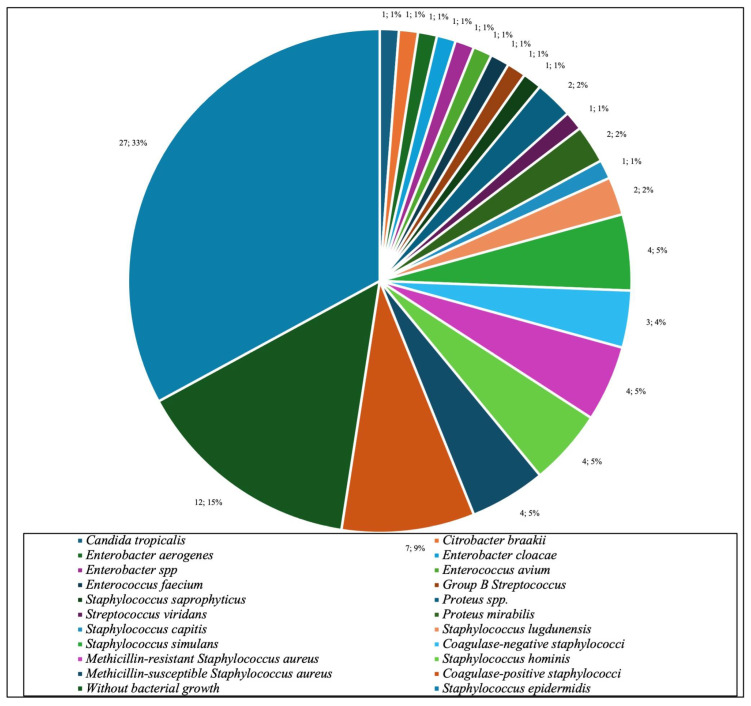
Isolated strains from chronic PJIs.

Figure A2 highlights the isolated strain from chronic cases of PJIs. Again, *Staphylococcus epidermidis* is the most frequent isolated microorganism, representing 33% of the cases (27 isolated strains), followed by culture-negative periprosthetic joint infections.

**Figure 4:**
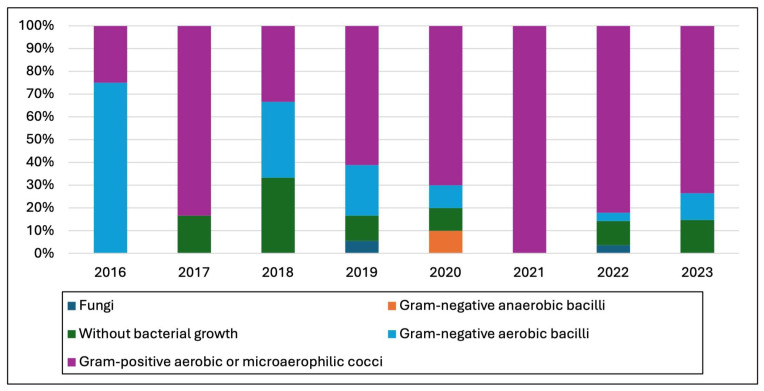
Patterns of isolated pathogens during the study period.

Figure 4 provides a graphical representation of the yearly breakdown and illustrates changes over time in the types of bacteria and fungi isolated from the harvested specimens, with a significant increase in the total cases from 4 in 2016 to 34 in 2023. These patterns, particularly the rise in Gram-positive aerobic or microaerophilic cocci cases, may be of interest for further epidemiological analysis or for guiding healthcare strategies regarding antibiotic perioperative prophylaxis.

### 3.3. Antimicrobial Susceptibility Test (AST) Results

**Figure 5:**
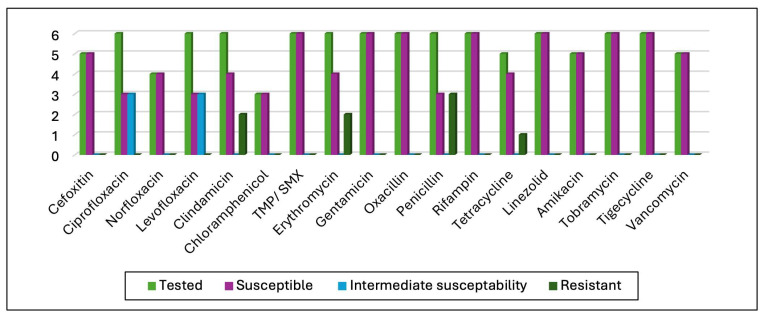
AST results for methicillin-susceptible *Staphylococcus aureus* isolates.

The study identified 55 coagulase-negative staphylococci (CoNS) strains, including 33 *Staphylococcus epidermidis*, with the rest comprising *S. lugdunensis, S. simulans*, *S. hominis*, *S. capitis*, *S. auricularis*, *S. saprophyticus*, and unidentified CoNS. Of these, 24 (43.63%) were methicillin-resistant, with 16 being *S. epidermidis*. Aminoglycoside testing (tobramycin, gentamycin, amikacin) showed 66% sensitivity to tobramycin (50 strains tested), 75.92% to gentamycin (54 strains), and 100% susceptibility to amikacin (54 strains). Penicillin G testing on seven strains found all resistant. Fluoroquinolone testing showed varying susceptibility: ciprofloxacin (17.31% susceptible, 63.46% intermediate, 19.23% resistant), levofloxacin (17.31% susceptible, 61.54% intermediate, 17.31% resistant), and norfloxacin (70% susceptible, 26.67% resistant). All strains were susceptible to linezolid, tigecycline, vancomycin, and rifampicin; 83.63% to clindamycin; 71.15% to tetracycline; and two strains resistant to trimethoprim/sulfamethoxazole. No vancomycin MIC exceeded 2 mg/L, indicating no reduced susceptibility encountered. Of 11 *Staphylococcus aureus* strains, 5 were methicillin-resistant, but all methicillin-susceptible strains remained responsive to a broad spectrum of antibiotics including second-generation cephalosporins, aminoglycosides, tigecycline, linezolid, trimethoprim/sulfamethoxazole, chloramphenicol, vancomycin, rifampicin, and norfloxacin, with ciprofloxacin and levofloxacin showing intermediate effectiveness against half of the strains without detected resistance. Further AST results are detailed in Figure 5.

**Figure 6:**
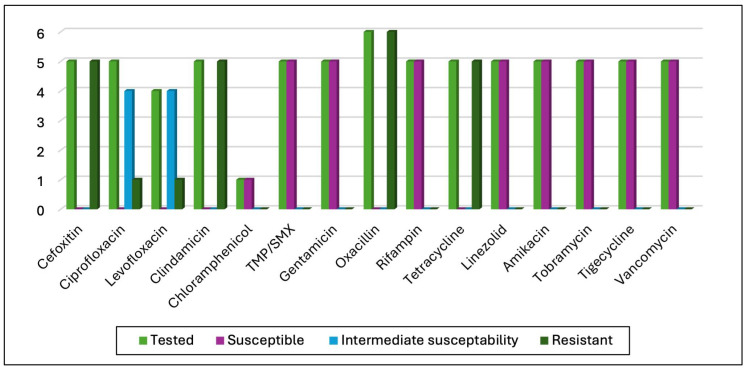
AST results for methicillin-resistant *Staphylococcus aureus* isolates.

All strains of Methicillin-resistant *S. aureus* maintained their susceptibility to aminoglycoside, tigecycline, linezolid, trimethoprim/sulfamethoxazole, vancomycin, and rifampicin. Ciprofloxacin and levofloxacin showed intermediate effectiveness against four of the tested strains, and resistance was detected in one tested strain. Details regarding the AST results are reported in Figure 6.

**Figure 7:**
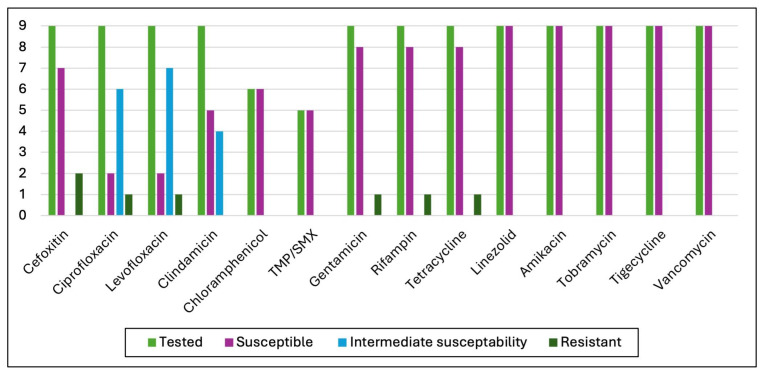
AST results of coagulase-positive staphylococci isolates.

Among nine coagulase-positive staphylococci strains, two were methicillin-resistant. Except for one strain resistant to gentamicin but susceptible to amikacin, all were susceptible to aminoglycosides, tigecycline, linezolid, trimethoprim/sulfamethoxazole, chloramphenicol, and vancomycin. No strain exhibited a vancomycin minimum inhibitory concentration (MIC) >2 mg/L. Moreover, eight strains showed susceptibility to rifampin, and two remained susceptible to ciprofloxacin and levofloxacin. For detailed Antimicrobial Susceptibility Test (AST) results, see Figure 7.

Given the limited isolation of Gram-negative bacilli and fungi strains in our study, we did not conduct a detailed analysis of the Antimicrobial Susceptibility Test (AST) and antifungal susceptibility results. Among the Gram-negative aerobic bacilli isolated, resistance profiles varied by species. All *Escherichia coli*, *Enterobacter* spp., and *Citrobacter freundii* strains were fully susceptible to all tested antibiotics. In contrast, resistance was observed in *Enterobacter aerogenes* to cefuroxime, levofloxacin, and piperacillin-tazobactam, and in *Citrobacter braakii* to cefuroxime. *Klebsiella oxytoca* exhibited the broadest resistance, with non-susceptibility to aminoglycosides, trimethoprim-sulfamethoxazole, fluoroquinolones, cefuroxime, ceftazidime, and amoxicillin-clavulanate. Resistance among *Proteus* species was heterogeneous: while some strains remained fully susceptible, others demonstrated resistance to ciprofloxacin, levofloxacin, trimethoprim-sulfamethoxazole, and cefuroxime. The strains of *Klebsiella oxytoca* and one strain of *Proteus mirabilis* demonstrated resistance to third-generation cephalosporins, particularly ceftazidime. These resistance profiles were phenotypically suggestive of extended-spectrum β-lactamase (ESBL) production. The *Pseudomonas aeruginosa* isolate showed intermediate susceptibility to aztreonam, imipenem, levofloxacin, and piperacillin-tazobactam, and resistance to ciprofloxacin. All Gram-negative isolates remained susceptible to meropenem.

The outcomes of the susceptibility tests are detailed in Table 5.

### 3.4. Multidrug-Resistant Periprosthetic Joint Infections

Our analysis of the crosstabulation matrix assessed the prevalence of multidrug-resistant (MDR) strains across all isolated microorganisms, following the definition by Magiorakos et al. [29] as acquired non-susceptibility to at least one agent in three or more antimicrobial categories. We identified 47 MDR strains and 49 non-MDR strains. Staphylococcus aureus and coagulase-positive staphylococci were notable for their high numbers of MDR strains, including five cases of methicillin-resistant *Staphylococcus aureus*, and five out of nine coagulase-positive staphylococci exhibiting MDR characteristics, respectively.

To ensure statistical accuracy, we excluded the two cases of PJI caused by *Candida* spp. and Gram-negative anaerobic bacilli (due to reasons already mentioned), respectively, with a focus on polymicrobial infections, cultures without bacterial growth, Gram-negative aerobic bacilli, and Gram-positive aerobic or microaerophilic cocci. Within these groups, 7 strains of Gram-negative aerobic bacilli (3 MDR) and 32 out of 73 strains of Gram-positive aerobic or microaerophilic cocci were MDR. Additionally, five out of seven polymicrobial infection strains were caused by multidrug-resistant organisms. Our statistical tests, including Pearson’s Chi-Square, Likelihood Ratio, and Linear-by-Linear Association, all indicated highly significant results (*p*-values < 0.001), highlighting a strong association between MDR strains and the type of microorganism.

**Figure A3:**
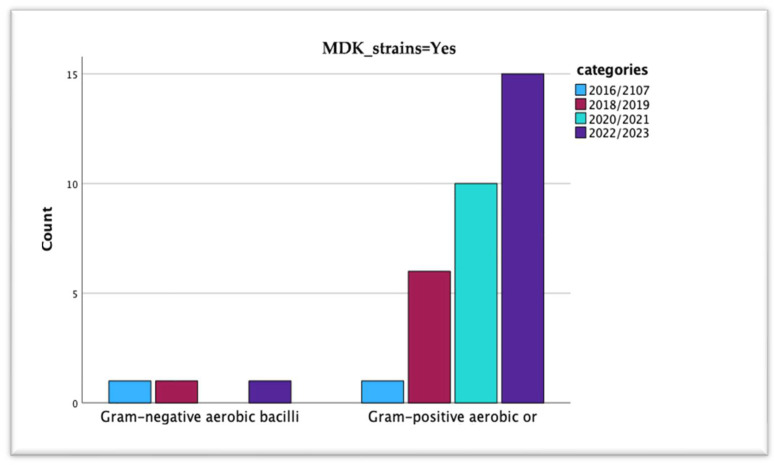
Patterns in the microbial etiology of PJIs during the study period by years in subcategories based on MDR status (MDR = Yes).

**Figure A4:**
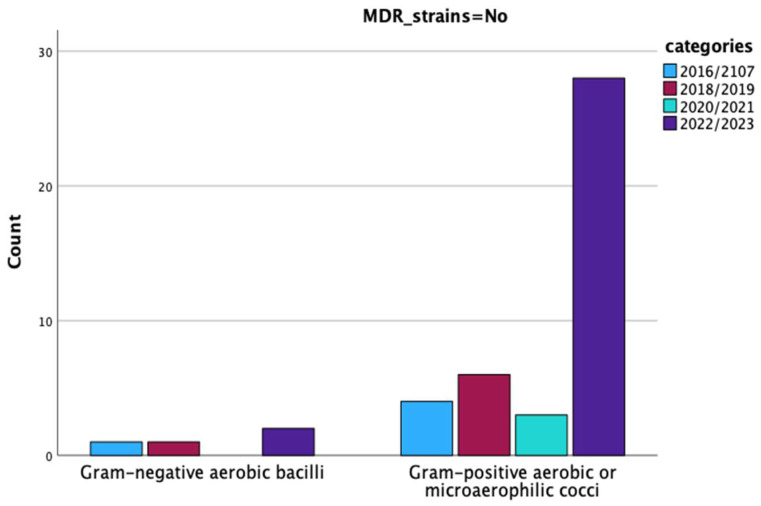
Patterns in the microbial etiology of PJIs during the study period by years in subcategories based on MDR status (MDR = No).

Further analysis on MDR strain distribution from 2016 to 2023, after excluding certain PJI cases, revealed no significant changes in the distribution of pathogen types or MDR strains over the periods from 2016/2017 to 2022/2023, as detailed in Table A2, and Figure A3 and Figure A4. The Chi-Square test results showed no significant association between infection type (acute or chronic) and the presence of MDR strains, with *p*-values > 0.05. This analysis, focusing on polymicrobial infections, Gram-negative aerobic bacilli, and Gram-positive aerobic or microaerophilic cocci, is reported in Table A3. Again, the Chi-Square test results showed no significant association between infection type (acute or chronic) and the presence of MDR strains, with *p*-values > 0.05.

Lastly, we investigated the potential association between the infection site (hip or knee) and MDR strain presence. No significant association emerged between the sample collection site and the presence of microorganisms, including MDR strains. This analysis involved polymicrobial infections, Gram-negative aerobic bacilli, and Gram-positive aerobic or microaerophilic cocci, with findings detailed in Table A4.

## 4. Discussion

Advancements in the preoperative diagnosis of PJI are crucial for managing these complications, yet diagnosing PJI remains a clinical challenge. Various guidelines from different societies, proposing diverse diagnostic criteria, aim to aid physicians in identifying patients with PJIs.

Empirical antimicrobial treatment, which involves addressing an infection without identifying the specific causative microorganism, targets the most probable microorganisms associated with the clinical scenario [30].

### 4.1. Pathogen Distribution and Epidemiological Patterns

Our results show that the choice of empirical antibiotic strategies in the case of acute PJIs should be focused on both Gram-positive aerobic or microaerophilic cocci and Gram-negative aerobic bacilli, mainly CoNS, Enterobacteriaceae, and *Staphylococcus aureus*, MDR strains, and possible polymicrobial infections. For chronic PJIs, empirical antibiotic treatment should also target CoNS, Enterobacteriaceae and *Staphylococcus aureus*, CoPS, MDR strains, and possible polymicrobial infections. Additionally, the high prevalence of culture-negative chronic PJIs (12 out of 13 cases) must be taken into consideration in the establishment of empirical antibiotic treatments.

Our results could inform the development of locally tailored empirical antimicrobial therapies, aiming to cover the most probable microorganisms while maintaining the narrowest possible antimicrobial spectrum.

As also reported by other authors [31], the most isolated microorganism in our study on periprosthetic joint infections belonged to the group of Gram-positive aerobic or microaerophilic cocci bacteria—CoNS (55 isolated strains), with *S. epidermidis* being the most common isolated species (33 out of the 55 strains of CoNS). Data from two specialist orthopedic hospitals in the United Kingdom highlight similar results, with the most frequent causative organisms being CoNS (137 of 384 – 36%) [32]. Comparable findings regarding microorganism profiles to ours and other published data were shown in New Zealand, where *Staphylococci* species (mainly *S. aureus* and CoNS) were the most common isolated organisms (52%) [23]. This aligns with many studies [22,33,34,35,36]. Fröschen FS et al. reported that the most frequent isolated pathogen in their study was CoNS at 44.9%, similar to ours (50.1%) [37,38,39]. Results from a multicenter study analyzing the leading microorganisms involved in PJI in China showed that the proportion of isolated strains belonging to the group of Gram-positive cocci was 65.7% [40]. Ma T et al. reported based on the results of their retrospective analysis of 76 patients diagnosed with PJIs that the most common isolated pathogens were staphylococci (61.3%) [41]. Benito N. et al. reported that the most commonly isolated causative microorganisms in their series of patients were also CoNS, with *Staphylococcus epidermidis* as the most frequent species [25]. They also noted that CoNS were more often isolated in chronic PJIs (>50% of cases) than in acute PJIs. Similar results were observed in our cohort of patients, where 76.36% of the isolated strains of CoNS were in chronic PJIs. Bacteria belonging to the CoNS group are part of the human skin microbiome and, despite their overall reduced virulence properties, account for most device-related infections due to their ability to form biofilms [42]. We found no significant difference in behaviors in our study. However, a percentage of CoNS was not identified at the species level in our study.

*S. aureus* was isolated in our study in 10.1% of the PJI cases in both acute and chronic PJIs, with six strains being MRSA strains. Other studies have reported a higher percentage of S. aureus isolates [25,43,44]. Fröschen FS et al. reported that in their study, S. aureus was isolated in 14.1%, similar to ours [37]. Wouthuyzen-Bakker M. et al. reported that 11.68% of the isolated strains of *S. aureus* were MRSA strains, results from a study on early acute and late acute PJIs treated with débridement, antibiotics, and implant retention [45]. CoPS were isolated in 9 (8.3%) cultures and were not identified to the species level in our study.

Previous data have shown that Gram-positive cocci are the most frequently isolated pathogens in cultures from cases with PJI. However, the literature is not always consistent regarding the role of CoNS and *S. aureus* [38,39]. While Tsai et al. and Mussa M et al. identified *S. aureus* as the most common isolated pathogen with an isolation rate of 26% and 27.5%, respectively, recent data published by Hu et al. and Stevoska et al. report CoNS with isolation rates of 38.1 up to 56.6% as more common and similar to ours [35,38,46,47]. Rondaan et al., in a recent publication on positive cultures strictly from sonication fluid, found that 50.5% of the isolated strains were CoNS, mainly *S. epidermidis* [48].

Overall, streptococci and enterococcus species accounted for only 1.8% and 2.7% of PJI cases in our study, respectively, and are more often involved in chronic periprosthetic joint infections. However, the literature reports vary in the involvement of these bacteria; streptococci are more commonly isolated in acute/acute hematogenous infections, while enterococci are more prevalent in chronic settings. Streptococci have been isolated in up to 30% of cultures, and enterococci in up to 13% [25,36,48,49].

Gram-negative aerobic bacilli were identified in 12.8% of cases, predominantly in chronic PJIs. This proportion is low compared to percentages observed in other recent studies (39–47%), which found most cases to be acute PJIs [22,25,50]. Similar to the classic series of PJIs, where Gram-negative aerobic bacilli account for less than 10% of cases [42], our findings suggest a lesser role for these bacteria. Despite this, the potential for such strains should not be overlooked. As reported by other authors [25], we did not find a statistically significant increased prevalence of Gram-negative bacilli in our study. Similar results were also reported in Romania by Roman M et al. [4], by McCulloch RA et al. in the United Kingdom [32], and by Fröschen FS et al. [37], underscoring the geographic variability and the complex nature of microbial profiles in PJI.

### 4.2. Multi-Drug Resistant Strains

Drug resistance is a complex phenomenon that arises from several factors. However, prolonged exposure to antibiotics is crucial leading to the selection of bacteria resistant to the related antibiotic. Moreover, hypermutable bacteria are more likely to acquire additional antibiotic resistance [32]. In our study, MDR strains, which primarily included MRSA and CoPS, were isolated in both acute and chronic PJI cases. Given that few previous studies have examined the involvement of MDR strains in the context of periprosthetic joint infections, our results should be interpreted with caution, as this is a single-center study. They should be corroborated by results from other studies. It is important to note that the epidemiology of MDR strains is likely to vary locally [4,25,51].

Nonetheless, our observations do not suggest that MDR PJIs are more commonly acute cases, contrary to what has been highlighted in previous reports [25]. Earlier studies have indicated that MDR organisms were more frequently isolated in early periprosthetic joint infections [25]. Casenaz A et al. reported that 29.1% of the infections were caused by MDR strains [52]. Roman M et al., reporting from another center in Romania, noted that 17.91% of cases were polymicrobial infections [4]. In our analysis, a concerning result emerged: 48.95% of the isolated strains were MDR strains.

### 4.3. Polymicrobial Infections

Polymicrobial PJI was defined as the detection of more than one microorganism in intraoperative cultures. Rates of up to 46.6% of polymicrobial infections have been reported in the literature [53,54]. Seven cases of PJIs were polymicrobial, and four of them were chronic cases of PJIs in our study. Fröschen FS et al. noted from a retrospective study on 493 PJI cases that 94 patients (19%) had polymicrobial PJIs [37]. Other authors also highlighted an increased number of polymicrobial PJIs, like Casenaz A et al. [52], who report that 29.1% (82/282) of the infections were polymicrobial ones, or Roman M et al. [4], in which 17.91% (12/67) of cases were polymicrobial infections [55]. Reduced rates of polymicrobial infections (7.8%) have been documented by Mussa M et al. Unlike other findings, we did not observe differences between acute and chronic polymicrobial PJIs [14,25].

In cases of acute PJIs, a wide range of bacterial species can be attributed to the likelihood of acquiring polymicrobial infections during surgery or wound healing. Also, based on the data published from the SKAR—Swedish knee arthroplasty register—polymicrobial PJIs are expected in early/acute PJIs [56]. Additionally, we found that CoNS were most isolated in both acute and chronic PJIs in both knees and hips. Similar data were published by Sebastian S et al. [57]. No differences in terms of the site of PJIs or type of PJIs by site were noticed in our study. This finding is consistent with previous studies [25,33,51,52]. Regarding chronic infections, some of the authors mentioned higher rates of polymicrobial infections (16.7%) compared to what is typically described in the literature [52]. Ravi S. et al. reported that in early PJIs, there is a rate of 42.85% polymicrobial infections (9/21) [58].

### 4.4. Antibiotic Susceptibility Test Results

To ensure the effective treatment of periprosthetic joint infection (PJI), pinpointing the responsible pathogen and conducting antibiotic susceptibility testing are critical steps. This process is crucial to targeted antibiotic therapy. Without identifying the specific pathogen, orthopedic surgeons must adhere to broad recommendations. Commonly, amoxicillin–sulbactam or amoxicillin–clavulanic acid is suggested as the initial therapy, although guidelines can vary internationally [59]. Thus, familiarity with the local microbiome is indispensable for selecting the optimal approach for both empirical and targeted treatments.

In managing PJIs, particular attention is drawn to the resistance patterns against specific antibiotics. Notably, a portion of Gram-negative aerobic bacilli showed resistance to ciprofloxacin (four strains—30.76%) and levofloxacin (five strains—38.46%). The growing resistance to quinolones is alarming, given their critical role in treating PJIs caused by isolated strains of Gram-negative bacilli [60]. Benito N et al. observed that nearly 18% of these bacilli are not susceptible to quinolone, indicating an uptrend in resistance [24]. Generally, wild-type strains of these bacilli are expected to be susceptible to fluoroquinolones like ciprofloxacin [61]. This was echoed by Fröschen FS et al., who reported comparable resistance rates to ciprofloxacin (17.3%, range: 0–35.71%) and piperacillin–tazobactam (26.5%, range: 0–57.1%), with no significant shifts in resistance patterns over time [39]. Our findings showed a modest reduction in piperacillin–tazobactam resistance (15.38%) among Gram-negative bacilli. Recent health advisories in countries such as Germany and Romania about ciprofloxacin’s side effects, including tendonitis and neuropathy, have led to restrained use, potentially curbing resistance. Moreover, Romania’s introduction of stricter antibiotic prescription policies in early 2024 aims to refine the selection of empirical antimicrobial therapy.

When PJIs are suspected to be caused by Gram-negative bacilli, considering piperacillin–tazobactam is prudent if there is a history of isolating such organisms. Despite resistance rates as high as 23.07% [39], it dose not remains a credible initial choice absent specific antibiotic susceptibility results. Currently, meropenem stands as a feasible first-line therapy within the local epidemiological setting, reserved for particular scenarios like septic shock or when alternative treatments are unfeasible. Caution is advised with its use to prevent escalating resistance. Notably, our isolates showed no resistance to meropenem.

Our analysis revealed that 24 of the isolated CoNS strains (43.63%) were methicillin-resistant, including 16 *S. epidermidis* strains. This pattern of oxacillin resistance, consistent with other studies [16], underscores the prominence of CoNS in PJI contexts. Remarkably, all strains were susceptible to rifampin, diverging from reports of notable rifampin resistance [16]. Moreover, *S. aureus* exhibited increased oxacillin resistance, with 5 out of 11 strains resistant, paralleling findings by Fröschen FS et al. [39]. This resistance trend was also observed in another Romanian center [4].

All isolated *S. aureus* strains remained susceptible to rifampin, and our study identified significant resistance to levofloxacin and ciprofloxacin, aligning with published data [62]. The critical role of CoNS in PJIs and associated adverse outcomes have been previously documented [63,64], highlighting the importance of considering vancomycin in antibiotic therapy before identifying the causative microorganisms, especially given the rare reports of vancomycin resistance among Gram-positive staphylococci. In our cohort, all strains were susceptible to vancomycin, indicating its potential effectiveness against *E. faecium* or *E. avium* strains. Although PJI with enterococci remains uncommon (2.3–15%), empirical therapy with beta-lactam antibiotics might not always be suitable [65]. In our study, enterococci were found in 1.8% of PJIs, with both strains susceptible to vancomycin, a finding that contrasts with reports of up to 19% PJI incidence caused by enterococci in other studies [39].

Moreover, all Gram-positive bacteria isolated in our study were susceptible to TMP/SMX, contrary to studies reporting up to 33.0% resistance [66]. This finding aligns with outcomes reported by Roman M. et al. [4]. Similar to other research [4,66], none of our CoNS, CoPS, or *S. aureus* strains showed resistance to linezolid, indicating its effectiveness. These findings emphasize the importance of local antimicrobial susceptibility data to guide empirical therapy, particularly in acute PJIs and data regarding multi-body-site colonization [67,68].

Considering the literature, culture-negative PJIs can account for 5% to 40% of cases, depending on the definitions and cohorts analyzed [69]. Our rate of culture-negative PJIs (11.9%) falls within this spectrum, underscoring a significant concern. Enhancing the routine diagnostic process and optimizing tissue sampling and handling can diminish the incidence of culture-negative findings. For instances where culture-negative PJI occurs, a variety of serological and molecular techniques stand available to assist in pinpointing the causative microorganism [69,70], ensuring that patients receive the most appropriate and effective treatment.

### 4.5. Patterns in Epidemiology or MDR-Isolated Strains

Our single-center study did not observe any statistically significant trends, either increasing or decreasing, in PJIs caused by Gram-positive aerobic or microaerophilic cocci or Gram-negative aerobic bacilli. This finding contrasts with Benito N et al.’s 2016 [24] report of an increase in PJIs due to aerobic Gram-negative bacilli [33]. Additionally, they noted a rise in fungal PJIs, whereas our study diagnosed two fungal cases. These results merit consideration within our study’s limitations.

To assess changes in causative microorganisms in PJIs, we conducted a biennial proportion analysis, revealing no significant shifts in microorganism distribution over time, aligning with findings by Roman M et al. [4]. This contrasts with Benito N et al.’s earlier observations [24] and their noted increase in fungal PJIs, diverging from our detection of two *Candida* spp. strains, resonating with Fröschen et al.’s findings of stable microorganism prevalence [37]. Over their six-year study, CoNS and S. aureus remained predominant, similar to Stefansdottir et al.’s observation of a rising trend in CoNS PJIs in Sweden from 1986 to 2000, alongside a decrease in *S. aureus* and enterococci [56]. However, Hu et al. described a slight decline in *S. aureus*, enterococci, and CoNS in China between 2006 and 2015 [25], contrasting with our findings and those of Benito N (2524 patients, 2003–2012) and Stefansdottir et al. (426 PJIs, 14 years) [24,47,56]. Hays M et al. [71] noted a significant increase in MRSA and a decrease in CoNS over 1990–2020, with no difference in MDR organisms by joint type, echoing our observations and Bjerke-Kroll et al.’s [72]. Unlike Hu L et al.’s report of rising methicillin/oxacillin resistance in *Staphylococcus* spp. [47], our data did not show a similar pattern.

Regarding empirical antimicrobial treatment for PJIs, we recommend coverage for staphylococci, including methicillin-resistant strains, Enterobacteriaceae, and *P. aeruginosa* for acute PJIs within the first month post-surgery. Options include glycopeptides or daptomycin, with the addition of an antipseudomonal cephalosporin like cefepime or ceftazidime. For chronic PJIs, empirical treatment should cover CoNS, methicillin-resistant staphylococci, and Enterobacteriaceae, with a third-generation non-antipseudomonal cephalosporin as an alternative to antipseudomonal agents. Empirical *Enterobacter* spp. coverage may also be necessary. Although rifampin and quinolones play a crucial role in PJI treatment, they should not be used initially. Alternative oral regimens might combine levofloxacin with clindamycin, minocycline, or co-trimoxazole, with a rifampicin and co-trimoxazole combination being effective against staphylococcal infections based on our findings. For Enterobacteriaceae infections, especially in cases of fluoroquinolone resistance, adverse reactions, or in the elderly, co-trimoxazole serves as an excellent oral alternative.

### 4.6. Implications for Antimicrobial Stewardship and Surveillance

The findings of this study underscore the importance of integrating local microbiological data into antimicrobial stewardship programs for infections including prosthetic joint infections. The observed rates of methicillin-resistant *Staphylococcus* spp. and ESBL-producing Gram-negative bacilli highlight the need for cautious empirical antibiotic selection, particularly in high-risk or referred cases. Empirical strategies for acute PJIs should prioritize coverage of Gram-positive cocci, particularly CoNS and *S. aureus*, with agents active against methicillin-resistant strains (e.g., vancomycin or linezolid), while also accounting for Gram-negative coverage in high-risk patients or polymicrobial infections. In chronic PJIs, the microbial spectrum is similarly broad and requires empiric regimens that balance efficacy with antimicrobial stewardship principles. Tailoring empirical therapy to local resistance patterns can reduce unnecessary broad-spectrum antibiotic use, improve patient outcomes, and slow the emergence of further resistance. Additionally, the presence of multidrug-resistant organisms across both acute and chronic PJIs reinforces the value of early diagnostics, including molecular testing when appropriate. Given the evolving nature of antimicrobial resistance, there is a clear need for sustained regional surveillance efforts. This is particularly critical in countries with limited published national data.

### 4.7. Limitations

Our study has some limitations, mainly related to its retrospective design. However, it would have been challenging to collect such a large number of prosthetic joint infection cases using any other design. Although the study assesses microbial etiology and patterns in one hospital, the number of enrolled patients did provide an overview of the local possible etiologies of PJI. Given its regional nature, these results should be interpreted in this note. Our use of multiple PJI definitions (Parvizi et al. pre-2022 [27], EBJIS post-2022 [26]) reflects real-world diagnostic transitions over time and was applied consistently based on the year of diagnosis. While this may introduce classification heterogeneity, core infection criteria remain largely overlapping. While our molecular assay (multiplex PCR with reverse hybridization) has shown high concordance with culture results in our experience, a formal validation against culture-positive samples was not performed within the scope of this study. Another limitation of this study is the absence of molecular testing for resistance genes, particularly those encoding extended-spectrum β-lactamases (e.g., blaCTX-M, blaSHV, blaTEM), in ceftazidime-resistant *Klebsiella oxytoca* and *Proteus mirabilis* strains. Although phenotypic profiles were indicative of ESBL production, the lack of genotypic confirmation limits the precision of resistance characterization. A key limitation of this study is the lack of normalization to hospital activity indicators such as admissions, surgical volume, or patient-days. As analyses were based on absolute case numbers, we cautiously used the term “patterns” rather than “trends” to describe microbiological and resistance findings over time. However, without denominator data, interpreting temporal changes remains limited—for example, stable MDR case numbers could reflect a relative increase if the overall patient volume decreased. Additionally, although some PJI cases were referred from other centers, this remains a single-center analysis.

Nevertheless, other studies in different parts of the world have shown comparable results in many respects. This study has several strengths that enhance the value of its findings. We have applied a consistent, standardized definition of multidrug-resistant organisms and included all consecutive patients diagnosed with a prosthetic joint infection, which helped minimize bias and ensured that different types of infection were adequately represented. By providing detailed microbiological data, we were able to highlight meaningful differences in the pathogens involved—information that can support more targeted empirical therapy and potentially improve patient outcomes. Although the data come from a single high-volume orthopedic center in Romania, they likely reflect a broader regional context based on a referral base. This referral pattern suggests that the microbial and resistance profiles described here are not limited to our institution alone. At the same time, national factors such as community antibiotic use and delays in referring complex cases may influence the patterns we observed. While our sample size is smaller than that of large multicenter studies, this work contributes valuable insights from a region where microbiological data on PJI remain scarce. These findings underline the importance of local surveillance efforts and the need for broader collaboration to better understand how PJI pathogens and resistance patterns vary across different healthcare settings.

## 5. Conclusions

Our study provides a detailed overview of the microbial etiology of prosthetic joint infections in a single-center cohort, contributing regionally relevant data to the broader understanding of PJI microbiology. The biennial analysis of isolated pathogens did not reveal significant changes in the distribution of microorganism types over the eight-year study period. Notably, our findings do not support the association of MDR PJIs with acute presentations, as suggested in prior reports. While temporal shifts may still occur in the future, our data reflect relative microbiological stability over the study period. For acute PJIs, empirical antibiotic therapy should primarily target Gram-positive cocci—particularly coagulase-negative staphylococci (CoNS) and *Staphylococcus aureus*—as well as Gram-negative bacilli, including Enterobacteriaceae and potential polymicrobial involvement. In chronic PJIs, similar microbial targets apply, with additional consideration for culture-negative infections. The observed resistance profiles, including the prevalence of methicillin-resistant staphylococci and suspected ESBL-producing Gram-negative bacilli, underscore the importance of tailoring antimicrobial regimens based on local susceptibility data. Ultimately, effective management of PJIs requires accurate microbiological diagnostics and a multidisciplinary approach to ensure timely and targeted therapy, especially in the context of emerging resistance patterns.

## Figures and Tables

**Table 1:** Baseline characteristics of patients diagnosed with PJI from 2016 through 2023.

Characteristic	No. of Cases (n = 102)
Median age (interquartile range, standard deviation), years	67.08 (11, ±8.848)
Male gender	53 (51.96)
Charlson Comorbidity Index, median (interquartile range)	3 (2)
Index arthroplasty site	
Hip	55
Knee	47
ASA score, median (interquartile range)	2 (1)
Indication for index arthroplasty	
Osteoarthritis	60 (58.82)
Avascular necrosis	9 (8.82)
Rheumatoid arthritis	4 (3.92)
Femoral neck fracture	12 (11.76)
other	17 (16.66)

If not otherwise stated, data are no. (%) of patients with indicated items.

**Table 2:** Causative microorganisms during the study period.

Microorganism or Group	Total no. of Positive Cultures n (%; 95% CI)
**Gram-positive aerobic or microaerophilic cocci**	80 (73.4; 65.1–81.7)
**CoNS—Coagulase-negative staphylococci**	55 (50.5; 42.2–59.6)
*Staphylococcus epidermidis*	33 (30.3; 22.0–39.4)
*Staphylococcus lugdunensis*	3 (2.8; 0.0–6.4)
*Staphylococcus simulans*	4 (3.7; 0.9–7.3)
*Staphylococcus hominis*	5 (4.6; 0.9–9.2)
*Staphylococcus capitis*	3 (2.8; 0.0–6.4)
*Staphylococcus auricularis*	1 (0.9; 0.0–2.8)
*Staphylococcus saprophyticus*	1 (0.9; 0.0–2.8)
**Unidentified CoNS**	5 (4.6; 0.9–9.2)
***Staphylococcus aureus***	11 (10.1; 4.6–16.5)
*Methicillin-resistant S. aureus*	5 (4.6; 0.9–9.2)
*Methicillin-susceptible S. aureus*	6 (5.5; 1.8–10.1)
***Streptococcus* species **	3 (2.8; 0.0–6.4)
Group B *Streptococcus*	1 (0.9; 0.0–2.8)
*Streptococcus viridans*	2 (1.8; 0.0–4.6)
***Enterococcus* species **	2 (1.8; 0.0–4.6)
*Enterococcus faecium*	1 (0.9; 0.0–2.8)
*Enterococcus avium*	1 (0.9; 0.0–2.8)
**CoPS—Coagulase-positive staphylococci**	9 (8.3; 3.7–13.8)
**Gram-negative aerobic bacilli**	14 (11.9; 7.2–19.3)
**Enterobacteriaceae**	12 (11.0; 3.7–15.5)
*Escherichia coli*	1 (0.9; 0.0–2.8)
*Enterobacter* spp.	3 (2.8; 0.0–6.4)
*Enterobacter cloacae* complex	1 (0.9; 0.0–2.8)
*Enterobacter aerogenes*	1 (0.9; 0.0–2.8)
*Enterobacter* spp.	1 (0.9; 0.0–2.8)
*Klebsiella oxytoca*	1 (0.9; 0.0–2.8)
*Proteus* spp.	2 (1.8; 0.0–4.6)
*Proteus mirabilis*	3 (2.8; 0.0–6.4)
*Citrobacter braakii*	1 (0.9; 0.0–2.8)
*Citrobacter freundii*	1 (0.9; 0.0–2.8)
**Gram-negative nonfermenting bacilli**	1 (0.9; 0.0–2.8)
***Pseudomonas* spp. **	1 (0.9; 0.0–2.8)
*Pseudomonas aeruginosa*	1 (0.9; 0.0–2.8)
**Gram-negative anaerobic bacilli**	1 (0.9; 0.0–2.8)
**Fungi**	2 (1.8; 0.0–4.6)
*Candida* spp.	2 (1.8; 0.0–4.6)
*Candida albicans*	1 (0.9; 0.0–2.8)
*Candida tropicalis*	1 (0.9; 0.0–2.8)
**Without bacterial growth**	13 (11.9; 5.5–18.3)

**Table 3:** Type of bacterial isolated strains during the study period.

		Years				Total
		2016/2107	2018/2019	2020/2021	2022/2023	
**Type of bacteria**	Without bacterial growth	1	3	1	8	13
	Gram-negative aerobic bacilli	2	2	0	3	7
	Gram-positive aerobic or microaerophilic cocci	5	12	13	43	73
	Polymicrobial	1	1	1	4	7
Total		9	18	15	58	100

Pearson Chi-Square value: 6.585; *p* = 0.680.

**Table 4:** Crosstabulation of isolated microorganisms by the type of PJIs (acute/chronic).

		Type of Infection *		Total
		Acute	Chronic	
Microorganisms	*Candida albicans*	1	0	1
	*Candida tropicalis*	0	1	1
	*Citrobacter braakii*	0	1	1
	*Citrobacter freundii*	1	0	1
	*E. coli*	1	0	1
	*Enterobacter aerogenes*	0	1	1
	*Enterobacter cloacae*	0	1	1
	*Enterobacter* spp.	0	1	1
	*Enterococcus avium*	0	1	1
	*Enterococcus faecium*	0	1	1
	Gram-negative anaerobic bacilli	1	0	1
	Group B *Streptococcus*	0	1	1
	*Klebsiella oxytoca*	1	0	1
	*Pseudomonas aeruginosa*	1	0	1
	*Staphylococcus auricularis*	1	0	1
	*Staphylococcus saprophyticus*	0	1	1
	*Proteus* spp.	0	2	2
	*Streptococcus viridans*	1	1	2
	*Proteus mirabilis*	1	2	3
	*Staphylococcus capitis*	2	1	3
	*Staphylococcus lugdunensis*	1	2	3
	*Staphylococcus simulans*	0	4	4
	Coagulase-negative staphylococci	2	3	5
	Methicillin-resistant *Staphylococcus aureus*	1	4	5
	*Staphylococcus hominis*	1	4	5
	Methicillin-susceptible *Staphylococcus aureus*	2	4	6
	Coagulase-positive staphylococci	2	7	9
	Without bacterial growth culture	1	12	13
	*Staphylococcus epidermidis*	6	27	33
Total		27	82	109

* Pearson Chi-Square value: 33.759; *p* = 0.209.

**Table 5:** Susceptibility test results for Gram-negative bacilli and *Candida* spp.

Bacterial Strain/Antibiotic (MIC/Result)	GEN	AMI	TOB	TMP/SMX	CRX	IMI	MEM	CIP	LFX	PPT	CTZ		
*Enterobacter cloacae*	S	S	S	S	R	S	S	S	S	S	S		
*Enterobacter* spp.	S	S	S	S	S	S	S	S	S	S	S		
*Enterobacter aerogenes*	S	S	R	S	R	I	S	S	R	R	R		
	**GEN**	**AMI**	**TOB**	**TMP/SMX**	**CRX**	**IMI**	**MEM**	**CIP**	**LFX**	**PPT**	**CTZ**	**CTR**	
*Citrobacter braakii*	S	S	S	S	R	S	S	S	S	S	S	-	
*Citrobacter freundii*	S	S	S	S	-	S	S	S	S	S	S	S	
	**GEN**	**AMI**	**TOB**	**TMP/SMX**	**CRX**	**IMI**	**MEM**	**CIP**	**LFX**	**NOR**	**PPT**	**CTZ**	**AMC**
*Klebsiella oxytoca*	R	R	R	R	R	S	S	R	R	R	S	R	R
	**GEN**	**AMI**	**TOB**	**TMP/SMX**	**CRX**	**IMI**	**MEM**	**CIP**	**LFX**	**NOR**	**PPT**	**CTZ**	**AMC**
*Proteus mirabilis*	R	S	R	R	R	I	S	R	R	R	I	R	R
*Proteus mirabilis*	S	S	S	S	S	S	S	S	S	S	S	S	S
*Proteus mirabilis*	S	S	S	S	S	I	S	S	S	S	S	S	S
*Proteus* spp.	S	S	S	S	S	S	S	S	S	S	S	S	R
*Proteus* spp.	S	S	R	S	S	S	S	R	R	R	S	S	S
	**GEN**	**AMI**	**TOB**	**TMP/SMX**	**CRX**	**IMI**	**MEM**	**CIP**	**LFX**	**NOR**	**PPT**	**CTZ**	**AMC**
*E. coli*	S	S	S	S	S	S	S	S	S	S	S	S	S
	**GEN**	**AMI**	**TOB**	**AZT**	**IMI**	**MEM**	**CIP**	**LFX**	**PPT**	**CTZ**	**COL**		
*Psuedomonas aeruginosa*	S	S	S	I	I	S	I	R	I	I	S		
*Candida* strain/Antimycotics	**5-FX**		**AmpB**		**FCZ**		**ITZ**		**VOR**				
*Candida albicans*	S		S		R		R		R				
*Candida tropicalis*	S		S		R		R		R				

Gentamicin—GEN; Amikacin—AMI; Tobramycin—TOB; Trimethoprim/Sulfamethoxazole—TMP/SMX; Cefuroxime—CRX; Imipenem—IMI; Meropenem—MEM; Ciprofloxacin—CIP; Levofloxacin—LFX; Piperacillin/tazobactam—PPT; Norfloxacin—NOR; Ceftazidime—CTZ; Ceftriaxone—CTR; Amoxicillin + clavulanic acid—AMC; Aztreonam—AZT; Colistin—COL; Flucytosine—5-FX; Amphotericin B—AmpB; Fluconazole—FCZ; Itraconazole—ITZ; Voriconazole—VOR.

## Data Availability

The original contributions presented in this study are included in the article. Further inquiries can be directed to the corresponding authors.

## Abbreviations

The following abbreviations are used in this manuscript:

PJI	Prosthetic joint infection
ASA score	American Society of Anesthesiologists score
MDR	Multidrug-resistant
CoPS CoNS	Coagulase-positive staphylococci Coagulase-negative staphylococci

## Appendix A

**Table A1:** Crosstabulation between isolated microorganisms by type of infection and study period.

Microorganisms × Type of Infection × Year Crosstabulation						Pearson Chi-Square Value	*p* =
Year			Type of Infection		Total		
			Acute	Chronic			
2016	Microorganisms	*Enterobacter aerogenes*	0	1	1	-	-
		*Proteus* spp.	0	2	2		
		Coagulase-negative *staphylococci*	0	1	1		
	Total		0	4	4		
2017	Microorganisms	Methicillin-susceptible *Staphylococcus aureus*	1	0	1	2.626	0.269
		Without bacterial growth	0	1	1		
		*Staphylococcus epidermidis*	1	3	4		
	Total		2	4	6		
2018	Microorganisms	*Enterobacter* spp.	0	1	1	3.000	0.223
		Methicillin-susceptible *Staphylococcus aureus*	1	0	1		
		Without bacterial growth	0	1	1		
	Total		1	2	3		
2019	Microorganisms	*Candida tropicalis*	0	1	1	9.775	0.369
		*E. coli*	1	0	1		
		*Enterococcus avium*	0	1	1		
		*Proteus mirabilis*	1	1	2		
		*Methicillin-resistant Staphylococcus aureus*	0	1	1		
		*Staphylococcus hominis*	1	0	1		
		Methicillin-susceptible *Staphylococcus aureus*	0	1	1		
		Coagulase-positive *staphylococci*	1	1	2		
		Without bacterial growth	1	1	2		
		*Staphylococcus epidermidis*	0	5	5		
	Total		5	12	17		
2020	Microorganisms	*Enterococcus faecium*	0	1	1	6.875	0.442
		Gram-negative anaerobic bacilli	1	0	1		
		Group B *Streptococcus*	0	1	1		
		*Streptococcus viridans*	0	1	1		
		*Proteus mirabilis*	0	1	1		
		Coagulase-positive *staphylococci*	0	2	2		
		Without bacterial growth	0	1	1		
		*Staphylococcus epidermidis*	1	1	2		
	Total		2	8	10		
2021	Microorganisms	Methicillin-resistant *Staphylococcus aureus*	0	1	1	2.917	0.405
		Methicillin-susceptible *Staphylococcus aureus*	0	1	1		
		Coagulase-positive *staphylococci*	1	1	2		
		*Staphylococcus epidermidis*	0	3	3		
	Total		1	6	7		
2022	Microorganisms	*Candida albicans*	1	0	1	17.395	0.097
		*Pseudomonas aeruginosa*	1	0	1		
		*Staphylococcus saprophyticus*	0	1	1		
		*Staphylococcus capitis*	0	1	1		
		*Staphylococcus lugdunensis*	0	1	1		
		*Staphylococcus simulans*	0	3	3		
		Coagulase-negative *staphylococci*	1	2	3		
		Methicillin-resistant *Staphylococcus aureus*	1	0	1		
		*Staphylococcus hominis*	0	1	1		
		Coagulase-positive *staphylococci*	0	3	3		
		Without bacterial growth	0	3	3		
		*Staphylococcus epidermidis*	1	8	9		
	Total		5	23	28		
2023	Microorganisms	*Citrobacter braakii*	0	1	1	22.120	0.076
		*Citrobacter freundii*	1	0	1		
		*Enterobacter cloacae*	0	1	1		
		*Klebsiella oxytoca*	1	0	1		
		*Staphylococcus auricularis*	1	0	1		
		*Streptococcus viridans*	1	0	1		
		*Staphylococcus capitis*	2	0	2		
		*Staphylococcus lugdunensis*	1	1	2		
		*Staphylococcus simulans*	0	1	1		
		Coagulase-negative *staphylococci*	1	0	1		
		Methicillin-resistant *Staphylococcus aureus*	0	2	2		
		*Staphylococcus hominis*	0	3	3		
		Methicillin-susceptible *Staphylococcus aureus*	0	2	2		
		Without bacterial growth	0	5	5		
		*Staphylococcus epidermidis*	3	7	10		
	Total		11	23	34		

Total: Pearson Chi-Square value: 33.759; *p* = 0.209.

**Table A2:** Crosstabulation between the type of isolated bacterial strains by years and by MDR status.

MDR Strains			Years				Total	Pearson Chi-Square Value	*p* =
			2016/2107	2018/2019	2020/2021	2022/2023			
No	Type of strain	Gram-negative aerobic bacilli	1	1	0	2	4	1.490	0.685
		Gram-positive aerobic or microaerophilic cocci	4	6	3	28	41		
	Total		5	7	3	30	45		
Yes	Type of strain	Gram-negative aerobic bacilli	1	1	0	1	3	5.719	0.126
		Gram-positive aerobic or microaerophilic cocci	1	6	10	15	32		
	Total		2	7	10	16	35		
Without bacterial growth	Type of strain	Without bacterial growth	1	3	1	8	13	-	-
	Total		1	3	1	8	13		
Total	Type of strain	Without bacterial growth	1	3	1	8	13	6.427	0.377
		Gram-negative aerobic bacilli	2	2	0	3	7		
		Gram-positive aerobic or microaerophilic cocci	5	12	13	43	73		
	Total		8	17	14	54	93		

**Table A3:** Crosstabulation between the type of isolated bacterial strains by years, by type of infections, and by MDR status.

MDR Strains			Type of Infection		Total	Pearson Chi-Square Value	*p* =
			Acute	Chronic			
No	Type_Gram_Fungi	Gram-negative aerobic bacilli	1	3	4	0.006	0.937
		Gram-positive aerobic or microaerophilic cocci	11	30	41		
	Total		12	33	45		
Yes	Type of microorganism	Gram-negative aerobic bacilli	1	2	3	0.365	0.546
		Gram-positive aerobic or microaerophilic cocci	6	26	32		
	Total		7	28	35		
Total	Type of microorganism	Gram-negative aerobic bacilli	2	5	7	0.098	0.754
		Gram-positive aerobic or microaerophilic cocci	17	56	73		
	Total		19	61	80		

**Table A4:** Crosstabulation between the type of isolated bacterial strains by years, by site, and by MDR status.

MDR Strains			Site		Total	Pearson Chi-Square Value	*p* =
			Hip	Knee			
No	Type of microorganism	Gram-negative aerobic bacilli	4	0	4	1.784	0.182
		Gram-positive aerobic or microaerophilic cocci	28	13	41		
	Total		32	13	45		
Yes	Type of microorganism	Gram-negative aerobic bacilli	1	2	3	0.122	0.727
		Gram-positive aerobic or microaerophilic cocci	14	18	32		
	Total		15	20	35		
Without bacterial growth	Type of microorganism	Without bacterial growth	5	8	13	-	-
	Total		5	8	13		
Total	Type of microorganism	Without bacterial growth	5	8	13	2.368	0.306
		Gram-negative aerobic bacilli	5	2	7		
		Gram-positive aerobic or microaerophilic cocci	42	31	73		
	Total		52	41	93		
